# Scleral Buckling for Rhegmatogenous Retinal Detachment Associated with Pars Planitis

**DOI:** 10.1155/2016/4538193

**Published:** 2016-09-05

**Authors:** Yong-Kyu Kim, Wontae Yoon, Jae Kyoun Ahn, Sung Pyo Park

**Affiliations:** ^1^Department of Ophthalmology, Kangdong Sacred Heart Hospital, Hallym University College of Medicine, Seoul, Republic of Korea; ^2^Jeonju Samsung Eye Clinic, Jeonju, Jeonrabuk-do, Republic of Korea

## Abstract

*Purpose*. To evaluate the surgical outcome of scleral buckling (SB) in rhegmatogenous retinal detachment (RRD) patients associated with pars planitis.* Methods*. Retrospective review of RRD patients (32 eyes of pars planitis RRD and 180 eyes of primary RRD) who underwent SB. We compared primary and final anatomical success rates and visual outcomes between two groups.* Results*. Primary and final anatomical success were achieved in 25 (78.1%) and 31 (96.8%) eyes in the pars planitis RRD group and in 167 eyes (92.7%) and 176 eyes (97.7%) in primary RRD group, respectively. Both groups showed significant visual improvement (*p* < 0.001) and there were no significant differences in final visual acuity. Pars planitis RRD group was associated with higher rate of postoperative proliferative vitreoretinopathy (PVR) development (12.5% versus 2.8%, *p* = 0.031). Pars planitis and high myopia were significant preoperative risk factors and pseudophakia was borderline risk for primary anatomical failure after adjusting for various clinical factors.* Conclusions*. Pars planitis associated RRD showed inferior primary anatomical outcome after SB due to postoperative PVR development. However, final anatomical and visual outcomes were favorable. RRD cases associated with pars planitis, high myopia, and pseudophakia might benefit from different surgical approaches, such as combined vitrectomy and SB.

## 1. Introduction

Pars planitis is a subset of intermediate uveitis characterized by the formation of snowbank or snowball in the absence of an associated infection or systemic disease [1]. In patients with pars planitis, the spectrum varies between asymptomatic patients at one end and the presence of progressive inflammation that leads to severe vision loss and complications at the other end. Common complications include cataract, epiretinal membrane, cystoid macular edema, and retinal neovascularization with or without vitreous hemorrhage [2, 3]. Retinal detachment (RD), a vision-threatening complication, occurs in up to 8.3% of pars planitis cases, and it is associated with exudation, traction, or rhegmatogenous causes [4–8].

Several surgical interventions are available for the treatment of rhegmatogenous retinal detachment (RRD) including scleral buckling (SB), pars plana vitrectomy (PPV), or a combination of both [9–13]. In phakic eyes, SB is a good treatment option for uncomplicated RRD as it has good visual outcome with lower cataract formation compared to PPV [9, 13]. In pseudophakic eyes, PPV showed good anatomical outcomes compared to SB [9]. In patients with RRD at high risk for postoperative proliferative vitreoretinopathy (PVR), PPV combined with SB showed higher rates of anatomical success compared with PPV alone [12].

The surgical outcomes of RRD associated with uveitis are known to be poor mainly due to frequent development of PVR after surgery [8]. However, specific information regarding anatomical and visual outcomes following SB in RRD associated with pars planitis is lacking. In this study, we aimed to compare the surgical outcomes of SB between patients with RRD associated with pars planitis and patients with primary RRD.

## 2. Material and Methods

We retrospectively reviewed the medical records of patients who underwent SB for RRD, either primary spontaneous RRD or secondary RRD associated with pars planitis, between January 1, 2005, and June 30, 2014, at our hospital. This study followed the tenets of the Declaration of Helsinki and was approved by the Institutional Review Board of Kangdong Sacred Heart Hospital. Pars planitis was diagnosed when characteristic snowbank or snowball and vitreous inflammation were observed without any evidence of infection or systemic disease. When RRD occurred in eyes with pars planitis, these were considered as secondary RRD associated with pars planitis. The following cases were excluded from the study: (1) trauma history; (2) epiretinal membrane or vitreomacular traction syndrome; (3) combined tractional RD; (4) PVR of grade B or greater; (5) diabetic retinopathy; (6) retinal vein occlusion; (7) previous intraocular surgery except uncomplicated cataract surgery; (8) postoperative follow-up < 12 months.

All surgeries were performed by one experienced retinal surgeon (SPP). All retinal breaks were identified and cryotherapy was performed during the surgery. Silicone sponge with width of 5 or 7.5 mm (number 506 or number 507, MIRA, Waltham, MA, USA) was used for the segmental SB, and silicone tire (number 287, MIRA, Waltham, MA, USA) and silicone band (number 240, MIRA, Waltham, MA, USA) were used for the encircling SB. External SRF drainage was performed during SB surgery in 30 of 212 cases (14.2%). In pars planitis cases, preoperative and postoperative systemic and topical steroid therapy were ensured for inflammation control. We prescribed oral prednisolone 40 to 60 mg/day (1 mg/kg body weight) for 1 week before the surgery and maintained the same regimen for two weeks after the surgery. Then, we gradually tapered it at a rate of 10 mg per week, considering the inflammatory status of the patients.

All patients underwent a thorough ophthalmologic examination, including best corrected visual acuity (BCVA; Snellen visual acuity chart), slit-lamp examination, and binocular indirect ophthalmoscopy. We converted Snellen visual acuity values into the logarithm of the minimal angle of resolution for the visual acuity analysis. Primary anatomic success was defined as complete retinal reattachment with one surgery and final anatomic success was defined as complete retinal reattachment following consecutive surgeries during the follow-up period. We compared visual outcomes and primary and final anatomic success rate between pars planitis associated RRD group and primary RRD group. We also compared baseline RD characteristics such as number or location of retinal breaks, RD extent, macula status (macula-on or macula-off), and postoperative PVR occurrence rate between two groups. Student's *t*-test was used for comparison of continuous variables and chi square test or Fisher's exact test was used for categorical variables. We also evaluated clinical factors associated with primary anatomic success by multiple logistic regression analysis. All statistical analyses were performed using PASW version 18.0 (SPSS, Inc., Chicago, Illinois, USA), and *p* values < 0.05 were considered statistically significant and *p* values ≥0.05 and <0.08 were considered borderline significant.

## 3. Results 

The 212 eyes (212 patients) that underwent SB for RRD were ultimately included; 32 eyes with pars planitis associated RRD and 180 eyes with primary spontaneous RRD. The baseline characteristics of the two groups are summarized in Table 1. Pars planitis RRD group was younger, male dominant and the proportion of pseudophakic eye was higher than primary RRD group. Pars planitis RRD group was also associated with greater number of retinal breaks and larger extent of RD compared to the primary RRD group. The proportion of round holes was high (62.5% versus 14.4%) and that of horseshoe tears was low (37.5% versus 85.6%) in pars planitis RRD group compared to primary RRD group. The proportion of high myopia, defined as spherical equivalent less than minus 6 diopters or axial length longer than 26 mm, was lower in pars planitis RRD group.

Surgical and visual outcomes are summarized in Table 2. Pars planitis RRD group underwent encircling SB more frequently than primary RRD group (50% versus 15.6%). Primary anatomic success rate was significantly lower in pars planitis RRD group (78.1% versus 92.7%, *p* = 0.017). Seven patients and 13 patients underwent subsequent PPV and intravitreal gas tamponade for recurring or nonresolving RRD in pars planitis RRD and primary RRD groups, respectively. After reoperation, 6 of 7 patients in pars planitis RRD group and 9 of 13 patients in primary RRD group showed reattachment of retina, while 1 and 4 patients in each group still showed persistent RD despite additional procedures. The overall final anatomic success rate was 96.8% in pars planitis RRD group and 97.7% in primary RRD group (*p* = 0.562). Pars planitis RRD group showed higher rate of postoperative PVR occurrence compared to primary RRD group (12.5% versus 2.8%, *p* = 0.031). Both groups showed significant visual improvement following surgery (*p* < 0.001 in both groups), and there were no significant differences in terms of baseline BCVA, final BCVA, and visual gain between two groups.

We evaluated preoperative clinical factors associated with primary anatomical failure following SB for RRD repair. Multiple logistic regression analysis was performed using the following parameters: pars planitis, high myopia, RD extent, number of retinal breaks, lens status (phakic versus pseudophakic), macula status (macula-on versus macula-off), and surgical method (segmental SB versus encircling SB). Presence of pars planitis (odds ratio 4.4, 95% confidence interval 1.4–13.9, and *p* = 0.012) and high myopia (spherical equivalent less than −6 diopters or axial length longer than 26 mm, odds ratio 3.4, 95% confidence interval 1.1–10.9, and *p* = 0.038) were significant factors associated with primary anatomical failure and pseudophakia (odds ratio 3.0, 95% confidence interval 0.9–9.7, and *p* = 0.070) showed borderline significance (Table 3).

## 4. Discussion 

Several factors must be considered when planning RRD treatment. When patient is young, SB is usually preferable compared to PPV for lessening the risk of postoperative cataract formation. However, in cases of RRD combined with PVR, PPV, either alone or combined with SB, might be a preferable choice. Previous reports on surgical outcomes of RRD repair are mainly done on low to medium complexity RRD cases [9, 10, 13–16], while surgical outcomes of uveitic RRD are scarce. It was reported that surgical outcome of RRD repair in uveitic patients is poor due to frequent development of PVR. However, uveitic RRD group in that study already showed higher rate of preoperative PVR and most common uveitis type that was associated with RRD development was panuveitis (6.6%). Only 4 out of 145 (2.8%) intermediate uveitis cases developed RRD [8]. Thus, in this study, we investigated whether SB is a good treatment option for RRD associated with pars planitis not accompanied by preoperative PVR.

The demographic and clinical characteristics of pars planitis associated RRD and primary RRD groups were different. The RRD associated with pars planitis was more prevalent in younger, male patients. The proportion of pseudophakic eye was high; however, that of high myopia was low in pars planitis RRD group. Pars planitis associated RRD showed large number of retinal breaks, which was mainly composed of round holes, and showed greater extent compared to primary RRD cases. Primary anatomic success rate was lower in pars planitis associated RRD group than primary RRD group (78.1% versus 92.7%). The main reason for this inferior surgical outcome seems to be associated with higher incidence of postoperative PVR development in this group. Four out of 32 patients (12.5%) developed postoperative PVR in pars planitis RRD group. This was lower than previously reported value in uveitic RRD cases (37%) [8] and, however, significantly higher than primary RRD group (2.8%). Although meticulous anti-inflammatory treatment was done during the perioperative period, postoperative PVR was more prevalent in pars planitis associated RRD group.

We also investigated preoperative risk factors associated with primary anatomical failure. Pars planitis itself was a significant risk factor for primary anatomical failure following SB surgery for RRD repair after adjusting for various clinical factors. High myopia was also a significant risk factor for primary anatomical failure and pseudophakia showed borderline significance. Interestingly, the proportion of high myopia in pars planitis RRD group was lower than that of primary RRD group. Myopia is closely related to the formation of atrophic holes and lattice degeneration [17], and myopia is a major risk factor of RRD in young adults [18–20]. In our cases, pars planitis RRD group showed higher rate of round holes than horseshoe tears despite lower incidence of high myopia, and it seems that round atrophic hole is also a characteristic of uveitic RRD [8]. Smith et al. reported that the most common form of retinal break found in intermediate uveitis is retinal hole due to vitreous tractions secondary to chronic vitreous inflammation [21]. A previous study reported that the intraocular inflammation in pars planitis damaged vitreous collagen structures and led to vitreous traction, possibly leading to multiple retinal holes, which is compatible with our results [22]. It is reported that PPV is more advantageous than SB in uncomplicated pseudophakic RRD cases, with less operating time, accurate diagnosis of breaks, and good anatomical outcome [9, 23, 24]. Our result also showed that pseudophakia is a clinical risk factor for primary anatomical failure following SB with borderline significance after adjusting other clinical factors.

It is unclear whether PPV is advantageous over SB in treatment of RRD associated with pars planitis from current study results. Storey et al. reported that, for patients at high risk for PVR, which was defined as RD extent of more than 2 quadrants, retinal tear >1 clock hour, preoperative PVR grades B-C, or a vitreous hemorrhage, combined PPV and SB were associated with significantly higher rate of anatomical success compared with PPV alone [12]. Pars planitis associated RRD cases in our study also share these high risk characteristics, and they might benefit from combined PPV and SB procedure. Unfortunately, currently, there are no effective surgical methods to prevent or treat PVR [25].

The final anatomical outcome and visual outcome of pars planitis RRD group were comparable with those of primary RRD group. This might be due to inclusion of only mild cases that were not accompanied by preoperative PVR grade B or C. Although primary surgical outcome following SB was inferior in pars planitis RRD group, recurring cases were well treated with additional PPV and gas tamponade. It might benefit from combined SB and PPV, particularly in pseudophakic cases.

This study is limited by its retrospective nature and by the relatively small numbers of patients. In addition, we only compared surgical outcomes of SB between pars planitis RRD and primary RRD groups and did not compare different surgical methods for treatment of pars planitis associated RRD. Further study might be needed for seeking optimal surgical method, principally to reduce postoperative PVR development.

In conclusion, RRD cases associated with pars planitis showed inferior primary anatomical outcome after SB due to postoperative PVR development, even in mild cases unassociated with preoperative PVR. However, final anatomical and visual outcomes were comparable with those of primary RRD cases. Pars planitis was an independent risk factor for primary anatomical failure following SB and primary vitrectomy with or without combined SB should be considered for high risk RRD cases, such as RRD associated with pars planitis.

## Figures and Tables

**Table 1:** Demographics and baseline characteristics of pars planitis associated rhegmatogenous retinal detachment (RRD) patients and primary RRD patients.

	Pars planitis RRD (*N* = 32)	Primary RRD (*N* = 180)	*p* value^a^
Age, yrs	39.1 ± 17.9	46.7 ± 19.1	0.037
Male, *n* (%)	23 (71.9)	95 (52.8)	0.045
Follow-up period, months	38.6 ± 17.5	36.1 ± 18.9	0.489
Pseudophakia, *n* (%)	10 (31.3)	22 (12.2)	0.013
High myopia^b^, *n* (%)	1 (3)	53 (29.4)	0.002
Number of retinal breaks ≥ 3, *n* (%)	16 (50)	30 (16.7)	<0.001
Type of retinal breaks			
Round holes	20 (62.5)	26 (14.4)	<0.001
Horseshoe tears	12 (37.5)	154 (85.6)	<0.001
Location of retinal breaks, *n* (%)			0.020
Superior	9 (28.1)	91 (50.6)	
Inferior	9 (28.1)	49 (27.2)	
Combined	14 (43.8)	40 (22.2)	
Macula-off RD, *n* (%)	7 (21.8)	71 (39.4)	0.058
RD extent > 6 o'clock, *n* (%)	27 (84.3)	112 (62.2)	0.017

^a^Student's *t*-test and chi square test or Fisher's exact test used for continuous and categorical variables, respectively.

^b^High myopia was defined as spherical equivalent less than −6 diopters or axial length longer than 26 mm.

RD: retinal detachment.

**Table 2:** Surgical and visual outcomes of pars planitis associated rhegmatogenous retinal detachment (RRD) patients and primary RRD patients.

	Pars planitis RRD (*N* = 32)	Primary RRD (*N* = 180)	*p* value^a^
Encircling, *n* (%)	16 (50)	28 (15.6)	<0.001
Subretinal fluid drainage, *n* (%)	4 (12.5)	26 (14.4)	0.513
Primary anatomic success, *n* (%)	25 (78.1)	167 (92.7)	0.017
Final anatomic success, *n* (%)	31 (96.8)	176 (97.7)	0.562
Postoperative PVR, *n* (%)	4 (12.5)	5 (2.8)	0.031
New or unfound retinal breaks, *n* (%)	3 (9.4)	8 (4.4)	0.220
Preoperative BCVA (logMAR)	1.13 ± 0.86	0.92 ± 0.89	0.228
Final BCVA (logMAR)	0.45 ± 0.51	0.31 ± 0.43	0.095
Visual gain (logMAR)	0.68 ± 0.64	0.61 ± 0.73	0.650

^a^Student's *t*-test and chi square test or Fisher's exact test used for continuous and categorical variables, respectively.

BCVA: best corrected visual acuity; logMAR: logarithm of the minimum angle of resolution; PVR: proliferative vitreoretinopathy.

**Table 3:** Preoperative clinical factors associated with primary anatomical failure after rhegmatogenous retinal detachment surgery with scleral buckling.

	Odds ratio	95% confidence interval	*p* value^a^
Pars planitis	4.4	1.4–13.9	0.012
High myopia^b^	3.4	1.1–10.9	0.038
Pseudophakia	3.0	0.9–9.7	0.070

^a^Multiple logistic regression analysis using backward elimination (*p* > 0.10) based on the probability of the likelihood-ratio.

^b^High myopia was defined as spherical equivalent less than −6 diopters or axial length longer than 26 mm.
